# Field Epidemiology and Public Health Research Priorities in the Eastern Mediterranean Region: Delphi Technique

**DOI:** 10.3389/fpubh.2021.690570

**Published:** 2021-12-03

**Authors:** Mohannad Al Nsour, Tala Chahien, Yousef Khader, Mirwais Amiri, Hana Taha

**Affiliations:** ^1^Global Health Development (GHD)|Eastern Mediterranean Public Health Network (EMPHNET), Amman, Jordan; ^2^Department of Community Medicine, Public Health and Family Medicine, Faculty of Medicine, Jordan University of Science & Technology, Irbid, Jordan; ^3^Basic Sciences Department, Faculty of Medicine, The Hashemite University, Zarqa, Jordan

**Keywords:** field epidemiology, public health, research priorities, Eastern Mediterranean Region, conflict countries

## Abstract

Research is essential for evidence-based decision making. This study aimed to identify research priorities in the areas of field epidemiology and public health in the Eastern Mediterranean Region (EMR) from the perspectives of public health professionals. A Delphi technique, using online survey, was employed to reach 168 public health professionals who have experience in the EMR countries. The study took place between November 2019 and January 2020. Consensus on the research priorities was reached after two-round online questionnaires. A list of top 10 field epidemiology and public health research priorities in the EMR was developed. Of those priorities, four fell under health in emergency, war and armed conflict, two under communicable diseases, two under immunization, one under digital health, and one under sexual, reproductive, and adolescent health. Availability, adequacy, and quality of health services in crisis settings were scored as a top priority (mean = 4.4, rank 1), followed by use of technology to improve the collection, documentation, and analysis of health data (mean = 4.28, rank 2), and capacity of countries in the region to respond to emergencies (mean = 4.25, rank 3). This study was conducted prior to COVID-19 pandemic and, thus, it did not capture COVID-19 research as a priority area. Nevertheless, identified priorities under communicable diseases including outbreak investigation of infectious diseases, epidemics and challenges related to communicable diseases in the EMR were still notable. In conclusion, the field epidemiology and public health research priorities identified in this study through a systematic inclusive process could be useful to make informed decisions and gear the research efforts to improve the health of people in the EMR.

## Introduction

Strengthening the field epidemiology and public health services in the Eastern Mediterranean Region (EMR) is a top priority nowadays due to several health challenges that resulted from political conflicts, environmental threats and natural disasters (1). The recent COVID-19 pandemic clearly revealed the fragility of health systems in many EMR countries, especially conflict-affected countries (2, 3).

Research is a vital tool that enables the improvement of health and health equity in low- and middle-income countries (LMICs), taking into consideration the critical needs and importance of sustainable development outcomes in these countries (4, 5). The 2013 World Health Report released by the World Health Organization (WHO) identifies four essential ways in which health systems can support health research: setting research priorities, building research capacity, defining norms and standards for research and translating evidence into practice (5). In LMICs, research should be prioritized to respond directly to community health needs. Otherwise, there is a risk that research conducted in LMICs will be according to funders' agendas, distort national priorities, undermine the role of national research in LMIC or fail to respond to explicit health needs (4, 6). If research priorities are not set, researchers and research sponsors cannot align their activities with national health and development goals (4).

Knowledge of the stage of health transition of a country and identifying diseases burden are necessary for effective setting of research priorities. Several studies have been conducted in LMICs to identify research priorities, including priorities in the fields of multisectoral collaboration for health (7), human resources for health (8), and adolescent health (9). A study conducted in 2019 that aimed to address primary care research priorities in 50 LMICs found four emerging priority areas: effective transition of primary and secondary services, horizontal integration within a multidisciplinary team and intersectoral referral, integration of private and public sectors, and ways to support successfully functioning PHC professionals (10).

There are several methods that are used for health research priority setting, including the 3-Dimensional Combined Approach Matrix (3D CAM) (11), the Child Health and Nutrition Research Initiative (CHNRI) method (12), the Council on Health Research and Development (COHRED) method (13), and the Delphi method (14). The Delphi method is based on a structured process for collecting and distilling knowledge from a group of public health professionals by means of a series of questionnaires interspersed with controlled opinion feedback. The aim of this process is to determine the future development of a particular topic by the public health professionals and help decision making on a profound basis. The features of the Delphi method include anonymity, iteration of arguments, communicativeness, well-founded feedback, wide range of expertise, rapidity of achieving consensus, and learning with dialogue (14). This technique has been extensively applied in research priority development and healthcare program planning (15).

This study aimed to identify research priorities in the areas of field epidemiology and public health in the EMR from the perspectives of public health professionals. The findings of this study are expected to help in decision making and planning of research resources, research funding, and implementation in the region to solve priority public health problems.

## Methods

Delphi technique was used in this study to reach an unanimity among public health professionals on field epidemiology and public health research priorities in the EMR Region. We employed this technique to instigate informational cascade from 168 public health professionals to reach a structured consensus. An experienced facilitator used a validated questionnaire followed by a prioritization tool employing a Likert scale (1–5) while conforming with the Delphi technique key features to reach consensus including: anonymity to avoid domination, private ranking in the group iteration that occurred in “rounds” and allowing the participants to change their minds. The consensus was reached after two rounds with controlled feedback.

We included a total of 168 public health professionals from all countries of the region representing different institutions, including ministries of health and academia, who were involved in Field Epidemiology Training Programs (FETPs), local and/or national level public health programs, and/or research entities in the EMR countries. These public health professionals were identified and selected from current Eastern Mediterranean Public Health Network (EMPHNET) databases, including its Regional Roster of Public health professionals and the EMPHNET Resources Engine (ERE). The public health professionals came from a wide range of background expertise and research interests in various public health areas. They all had leading/technical positions in their relevant institutions which contributed to the identification of the priority research areas during the Delphi process. They served as individuals with expert opinion about the subject matter and key informants to the prioritization process.

This research project comprised of a two-round online questionnaire. A facilitator familiar with the Delphi technique facilitated the process of identifying the focal points for FETPs recruiting public health professionals and following up with them in both rounds. The main language used throughout the process (both rounds) was English. However, some initial inputs for the first round were also provided in Arabic and were translated to English. Both rounds took place between November 2019 and January 2020.

The first-round questionnaire was developed using the KoBo Toolbox platform and sent to public health professionals by email. It collected general demographic details including years of experience. Each expert was then requested to identify up to five specific priority research questions in the area of field Epidemiology and/or public health in their respective countries or in the EMR region. They were also asked if they were willing to receive the second-round questionnaire to prioritize research areas. The median duration spent on filling out the first-round questionnaire was 17 min, with at least one third of the participants filling out the questionnaires in 6 to 15 min.

The research questions raised by the public health professionals in round one were then synthesized, clustered, and grouped into 11 major areas: (1) Mental health, (2) Sexual, reproductive health and adolescent health, (3) Communicable diseases, (4) Non-communicable diseases, (5) Health in Emergency, War and Armed Conflict, (6) Immunization/vaccination, (7) Disease surveillance, (8) Field Epidemiology Training Program (FETP), (9) Health care and health system, (10). Health technology, and (11) Zoonotic diseases, with research topics under each area.

**Figure 1:**
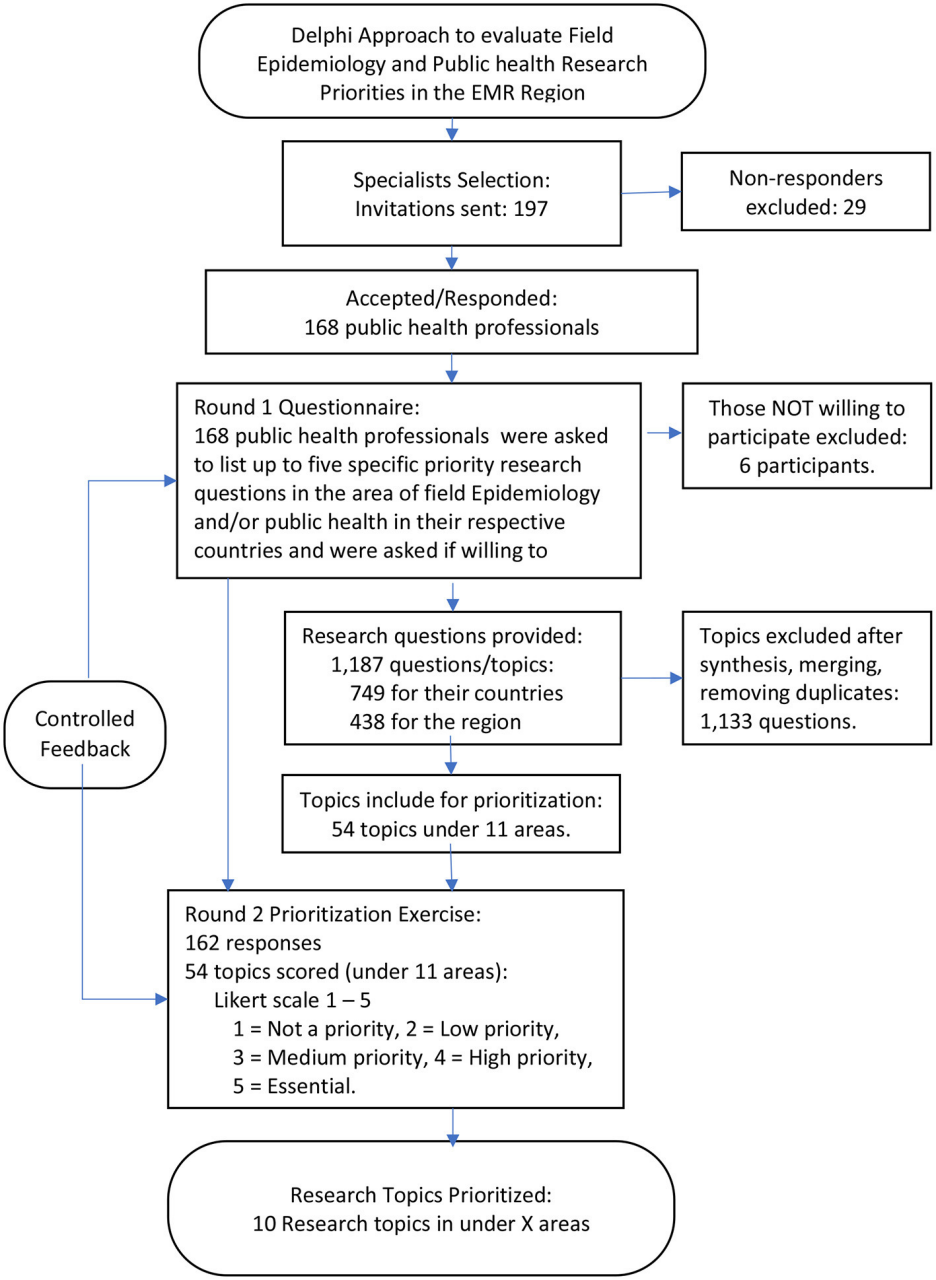
The Delphi process to evaluate research priorities in the EMR.

The results obtained from this questionnaire (i.e., the research topics/questions under each) were analyzed to develop the second-round questionnaire. The second online survey that included a battery of research topics (for countries and for the region) was then sent using the same platform and only to those who agreed to be contacted again. At this stage, participating public health professionals were asked to score/rank the research topics using five-point Likert scale; 1—Not a priority, 2—Low priority, 3—Medium priority, 4—High priority, and 5—Essential. Respondents were requested to rate each research topic. The responses from the second questionnaire were analyzed using the average scores from the above ranking. In addition to calculation of mean priority score, the percentages of public health professionals who rated the research topic as essential were calculated. To conclude, the analysis was shared with the public health professionals to standardize their responses. The overall process is summarized in Figure 1.

## Results

### Participants' Characteristics

A total of 168 public health professionals (112 males and 56 females) from all EMR countries responded to first round questionnaire. Their average years of experience after graduation from the highest degree they earned was 9.7 years. Of the total health professionals, 67 (39.9%) were from ministries of health, 54 (32.1%) from academia, 25 (14.9%) from FETPs, 12 (7.1%) from local and/or national level public health programs, and 10 (6.0%) from research entities in the EMR countries.

### Round 1

A total of 1,187 research questions were raised by the public health professionals. After removing duplicates and repetitive questions, the remaining questions (425 questions) were synthesized by merging similar questions and coming with broader research topics. Finally, a total of 54 research topics were clustered and grouped into 11 major areas. The 54 research topics were then presented and used in the second-round questionnaire to be ranked.

### Round 2

Table 1 shows the priority scoring of 54 research topics categorized according to research area. Of the top 10 priority areas identified according to the mean priority score: four priority areas fell under Health in Emergency, War and Armed Conflict, two under communicable diseases, two under immunization, one under digital health, and one under sexual, reproductive, and adolescent health. Table 2 shows the top 10 priority research areas. Availability, adequacy, and quality of health services in crisis settings were scored as a top priority (mean = 4.4, rank 1), followed by the use of technology to improve the collection, documentation and analysis of health data (mean = 4.28, rank 2), and capacity of countries in the region to respond to emergencies (mean = 4.25, rank 3). The other research topics identified as among top 10 priority topics were outbreak investigation of infectious, causes of maternal and infant mortality, state of preparedness in the region to address a disease outbreak, epidemics and challenges related to communicable diseases in the EMR Region, situation of health during crisis/emergencies in the EMR region, risk assessment of emergencies and threats in the EMR Region, and immunization gaps in the region identified through disease surveillance and regional response guidelines.

**Table 1:** The priority scoring of 54 research topics categorized according to research area.

**Research Areas and Research Topics**	**Priority average score**		**Scored as “essential”**	
	**Mean**	**SD**	**n**	**%**
**Mental health**				
Mental health of refugees, particularly children and adolescents	3.83	1.08	19	31.7
Promotion and protection of the rights of people with mental disorders	3.56	0.82	6	10.0
Mental health promotion and advocacy	3.58	0.81	6	10.0
Evaluation of mental health programs	3.48	0.91	8	13.3
**Sexual, reproductive, and adolescent health**				
Causes of maternal and infant mortality	4.22	0.98	30	50.0
Importance of enforcing and implementing public health legislation to reduce morbidity and mortality in the EMR	4.08	0.89	22	36.7
Sexual transmitted diseases	3.82	0.87	15	25.0
Integrating Minimum Initial Service Package (MISP) into primary healthcare	3.78	1.03	17	28.3
Sexual Based Violence (SBV) and Gender Based Violence (GBV)	3.67	0.88	10	16.7
Pattern of domestic violence	3.32	0.85	4	6.7
Burden, leading causes and treatment of infertility	3.18	0.98	6	10.0
**Zoonotic diseases**				
Opportunities and challenges of zoonotic disease surveillance and one health approach	4.03	0.82	18	30.0
Managing, treating and preventing common zoonotic diseases in the EMR (i.e., brucellosis, rabies, hemorrhagic fever)	3.93	0.92	19	31.7
Climate change and the emergence of vector-borne diseases	3.82	0.97	17	28.3
Impact of war on the emergence of zoonotic disease	3.68	1.09	12	20.0
**Communicable disease**				
Outbreak investigation of infectious diseases (H1N1, Cholera, measles, polio, brucellosis)	4.23	0.70	22	36.7
Epidemics and challenges related to communicable diseases in the EMR region	4.20	0.71	22	36.7
Real burden of Anti-Microbial Resistance (AMR), microorganisms of concern, and interventions to manage/prevent AMR	4.07	0.78	19	31.7
Burden, management and treatment of MERS-CoV in the EMR region	4.02	0.87	21	35.0
Effect of armed conflicts in the region on the map of infectious diseases	3.92	1.06	20	33.3
Hospital acquired infections	3.92	0.77	13	21.7
Mathematical modeling to control emerging infectious diseases	3.75	0.84	10	16.7
The pattern of diseases encountered among pilgrims	3.68	0.89	11	18.3
**Research topics related to Non-Communicable Diseases (NCDs)**				
Burden, challenges, prevention, control of Non-Communicable Diseases in the region (Cardiovascular diseases, obesity, diabetes)	4.08	0.81	20	33.3
Effect of smoking cigarettes/ e-cigarettes on health	3.65	1.09	17	28.3
The determinants of uncontrolled obesity and how to overcome it	3.58	0.98	11	18.3
**Health in Emergency, War and Armed Conflict**				
Availability, adequacy and quality of health services in crisis settings	4.40	0.69	30	50.0
Capacity of countries to respond to emergencies	4.25	0.75	25	41.7
State of preparedness to address a disease outbreak	4.22	0.72	22	36.7
Situation of health during crisis/emergencies in the EMR region	4.20	0.75	22	36.7
Development of interventions to improve the health of vulnerable populations	4.03	0.66	13	21.7
Assessment of health systems and health-related interventions in War and Armed Conflict areas	4.02	1.03	21	35.0
Health of refugees/conflict related victims and the quality of services delivered to this population	3.88	0.87	13	21.7
Association between conflicts/wars and emergence of diseases	3.77	0.93	10	16.7
Supporting sustainable development and community-based assets in fragile settings	3.76	0.86	11	18.3
**Research topics related to Immunization/vaccination**				
Immunization gaps in the region identified through disease surveillance and regional response guidelines	4.10	0.84	21	35.0
Re-emergence of diseases (i.e., measles) despite vaccination efforts	4.05	0.83	18	30.0
Impact of vaccination on human health / reduction of diseases	3.95	1.07	24	40.0
Impact of Expanded Program on Immunization (EPI) coverage	3.95	0.91	19	31.7
**Disease Surveillance**				
Risk assessment of emergencies and threats	4.15	0.68	18	30.0
Initiatives to strengthen priority disease surveillance	4.08	0.65	15	25.0
Utilization of innovative tools/ research for evolving Public Health Surveillance	4.00	0.76	15	25.0
Level of networking and collaboration between stakeholders during outbreaks in the EMR region	3.98	0.75	15	25.0
**Research topics related to Field Epidemiology Training Program (FETP)**				
Impact of Field Epidemiology Training Programs on the timeliness of disease surveillance, detection and response	4.08	1.01	25	41.7
Field screening to determine disease outbreak in the region (i.e., vector-borne outbreaks)	3.98	0.83	17	28.3
**Health care and health system**				
Challenges in ensuring data quality of health information system and utilization of data for policy action	4.07	0.67	15	25.0
Strategies to improve the health system in the region.	4.07	0.69	14	23.3
Challenges of achieving Universal Health Coverage (UHC) in the region	4.05	0.77	18	30.0
Allocating and utilizing resources to improve the health of the community	4.05	0.62	13	21.7
Rebuilding Health Systems in Fragile and Conflict Affected States	3.95	0.84	13	21.7
The effect of social/cultural factors on shaping the health seeking behavior	3.73	0.81	7	11.7
Human right	3.67	1.08	15	25.0
**Digital health**				
Use of technology to improve the collection, documentation and analysis of health data	4.28	0.72	25	41.7
Developing national electronic health records	4.02	0.83	18	30.0

**Table 2:** The top 10 priority research topics in public health and field epidemiology.

**Research areas**	**Priority research topics**	**Mean priority score**	**Rank**	**Percentage scored essential**	**Rank**
Health in Emergency, War and Armed Conflict	Availability, adequacy, and quality of health services in crisis settings	4.4	1	50	1
Digital health	Use of technology to improve the collection, documentation and analysis of health data	4.28	2	41.7	3
Health in Emergency, War and Armed Conflict	Capacity of countries in the region to respond to emergencies	4.25	3	41.7	4
Communicable disease	Outbreak investigation of infectious diseases (H1N1, Cholera, measles, polio, brucellosis)	4.23	4	36.7	7
Sexual, reproductive, and adolescent health	Causes of maternal and infant mortality	4.22	5	50	2
Health in Emergency, War and Armed Conflict	State of preparedness in the region to address a disease outbreak	4.22	6	36.7	8
Communicable disease	Epidemics and challenges related to communicable diseases in the EMR	4.2	7	36.7	9
Health in Emergency, War and Armed Conflict	Situation of health during crisis/emergencies in the EMR	4.2	8	36.7	10
Disease surveillance	Risk assessment of emergencies and threats in the EMR	4.15	9	30	21
Immunization/vaccination	Immunization gaps in the region identified through disease surveillance and regional response guidelines	4.1	10	35	12

Using the percentage of respondents who identified the research area as essential, two areas belonging to immunization and surveillance were identified as of less priority. Instead, the impact of Field Epidemiology Training Programs on the timeliness of disease surveillance, detection, and response (percentage = 41.7%, rank = 5) and impact of vaccination on human health/reduction of diseases (percentage = 40.0%, rank = 6) were identified among the top 10 research priorities.

## Discussion

Of the 10 priorities, four fell under health in emergency, war and armed conflict. This finding may be explained by the fact that, in the past years, the Region has been affected most by emergencies, war, and armed conflict. Most of the field epidemiology staff in these countries are dealing with issues caused by these crises, including the issues related to the availability, adequacy, and quality of health services, workforce capacity and preparedness to respond to crises and disease outbreaks, and any assessments needed during crises/emergencies. However, this study still represents a wide view of public health professionals regarding the field epidemiology and public health in the EMR Region. In terms of practicality of the approach, the median duration for filling out first round questionnaires was 17 min, which provides insights into planning for questionnaires of a similar length for distribution to a large list-serve/roster of public health professionals and getting back valuable inputs in a reasonably short period of time. This study was conducted before the COVID-19 pandemic. Thus, the questionnaires did not specifically ask for and capture any prioritization of COVID-19 research. However, although not mentioned directly in the collected questions, public health professionals provided six questions about MERS-CoV and one on SARS in the collected data, indicating the importance of this family of viruses even before the COVID-19 pandemic. These questions focused on the prevalence of SARS and MERS-CoV in the EMR region, risk of travel associated cases of MERS CoV, exploring the difference of MERS CoV strain of viruses from one country to another, and the role of cultural and religious leaders in controlling MERS-CoV.

The top research priority was focused on the availability, adequacy, and quality of health services in crisis settings (emergency, war and armed conflict). The EMR is afflicted with several foci of humanitarian crises and civil unrest, including the Syrian, Yemeni, Libyan, Iraqi, Palestinian and Sudanese crises. Following the Syrian crisis in 2011, approximately 6.6 million Syrians were internally displaced, and around 5.6 million sought refuge in surrounding countries (16). As for the Yemeni crisis in 2011, more than 2 million people were displaced, and 14 million are now facing pre-famine conditions (17). The region is facing many health problems, including the re-emergence of communicable diseases such as polio and malaria, in addition to non-communicable diseases such as malnutrition, and post-traumatic stress disorder (PTSD).

In areas of conflict, research is not prioritized, as resources are directed toward security, conflict resolution, acute humanitarian response, and refugee/ migration management. A study conducted in Lebanon, a country with a long history of political conflicts and a home to many refugees, showed that there is a lack of nationwide research culture, health researchers facing insufficient funding and limited access to data, insufficient training of medical students to conduct research and poor impact of research on policy (18). However, research capacity strengthening in conflict settings aids in collecting highest standard evidence, assessing health needs of affected population, bridging the gap between research and practice, and eventually, informing advocacy and policy change. Furthermore, strengthening research capacity may help address major endemic diseases, evaluate and improve relief work, and support social changes to improve the quality of assistance provided.

Use of technology to improve the collection, documentation and analysis of health data was ranked the second top research priority. In many countries in the EMR, health informatics governance is very weak due to a lack of national strategies and policies, or non-adherence to them, leading to a fragmented health information development and implementation. The public are not involved in policy development, monitoring and accountability for health information technology, causing lack of confidentiality and uncertainty regarding data ownership. Furthermore, integrated health informatics education programs are scarce in the region (19).

Ranked third and fourth top priority research topics were capacity of countries in the region to respond to emergencies and outbreak investigation of infectious diseases. Countries in the EMR are a hotspot for emerging and reemerging infectious diseases. In the past decade, Sudan has experienced 3 outbreaks of Yellow fever, and Bahrain, Egypt, Iran, Jordan, Kuwait, Lebanon, Oman, Qatar, Saudi Arabia, Tunisia, the United Arab Emirates and Yemen faced the Middle East Respiratory Syndrome, among several other widespread outbreaks in the EMR, such as Chikungunya, Cholera, and influenza A (H1N1, H5N1, and H9N2) (20). Furthermore, internally displaced people, refugees and host communities are at a high risk of potential outbreaks due to fragile public health systems and weak surveillance and detection.

Low levels of awareness concerning infection symptoms at the early stages of the disease, especially at the primary care level and in hospital departments for NCDs, leads to clinical diagnostic mistakes, and poor management of infection control measures. The regional preparedness, response and control efforts toward communicable diseases face major challenges such as poor surveillance, lack of integrated vector management approaches, week multidisciplinary and intersectoral collaboration, and the absence of comprehensive readiness and response plans (21). Moreover, there is a need for consensus for establishing a standard case definition for infections to prevent major gaps in definition and reporting of mortality rates between endemic countries.

The fifth top research priority was causes of maternal and infant mortality. Maternal mortality ratio (MMR) is one of the main criteria of health outcomes and an indicator of socioeconomic development. In the EMR Region, except in Djibouti, Palestine and Afghanistan, maternal mortality from 1990 to 2015 has declined, but the burden remains higher than the global average (22). A study that aimed to prioritize research on sexual and reproductive health in the African and EMR found that one of the priorities is addressing adolescent violence and preventing early pregnancy, as early marriage and pregnancy leads to higher maternal and infant mortality rates (23). Morocco and Jordan have implemented interventions that have drastically reduced maternal mortality by >75% from the year 1990 to 2015, while low-income countries have seen minimal to no improvement (22). Some challenges to improving maternal health include manmade disasters and conflicts, political instability, domestic crises, and economic sanctions (22, 24).

Epidemics and challenges related to communicable diseases, health during crisis/emergencies, risk assessment of emergencies and threats and immunization gaps were ranked among the top 10 priority research topics. The EMR has seen a great improvement in routine vaccination coverage, but political unrest, complex emergencies, humanitarian crises and socioeconomic hardship affecting the region have caused disruption of immunization systems in several countries. For example, the coverage of the Diphtheria, Tetanus and Pertussis vaccine and third dose of the oral polio vaccine in the region has dropped form 86% in 2010 to 80% in 2015, with wide inter-and intra-country differences (25).

There are many disparities in influenza vaccination rates between different countries in the EMR, as well as low vaccination coverage. A study that investigated seasonal influenza vaccination policies in the EMR showed that of the 20 countries involved in the study, only 14 had seasonal influenza vaccination policies at the time of the survey, and of these 14, only five included the vaccine in their national immunization program (26). Furthermore, of the 20 countries, three countries did not have an active influenza surveillance system.

One of the strengths of this research approach is the convenience and the rapid consensus that was achieved in two rounds. The whole process was virtual without having the participants in the same room to get the group dynamics and reach agreement. We were able to gather wide range of public health professionals from all countries of EMR at a relatively low cost of administration and analysis. The respondents were free to express their opinion against the group thinking. The Delphi technique was useful to gain valuable insightful data about a topic where knowledge was lacking.

### Limitations

Like every other study, this one has its limitations because the Delphi technique does not cope well with widely differing opinions or paradigm shifts. Also, there may be chances of selection bias involved because participation was voluntary and those who responded to the two rounds of questionnaires might be different from those who did not respond. However, an explanation of the four priorities linked with public health emergencies can be found at the beginning of the Discussion section. Also, the existence of any selection bias could not be detected through the implemented technique and, given the qualitative nature of the collected information, any mechanism to look for such bias would have little effect on the type of questions that were generated by subject matter experts in the first round and ranked in the second round.

## Conclusions

The results of our study highlight the importance of training future researchers in a contextualized approach toward field epidemiology and public health in conflict settings. Furthermore, there is a need to improve and expand health informatics training to increase the number of health information technology professionals. Policymakers should be involved in designing research agendas, and this can be achieved by multiple stakeholder involvement and collaboration. Strategic frameworks should be developed for prevention and control of emerging diseases, and for integrating early warning systems for diseases outbreaks in countries affected by humanitarian crises. Moreover, there is a need for establishing standard case definitions for infections, and developing an algorithm for clinical, epidemiological, occupational, and other demographic characteristics.

## Data Availability Statement

The raw data supporting the conclusions of this article will be made available by the authors, without undue reservation.

## Ethics Statement

The studies involving human participants were reviewed and approved by the Institutional Review Board of Jordan University of Science and Technology. Written informed consent for participation was not required for this study in accordance with the national legislation and the institutional requirements.

## Author Contributions

MAN, TC, and YK contributed to conception, design of the study, manuscript revision, and wrote the first draft of the manuscript. YK performed the statistical analysis. HT wrote sections of the manuscript and contributed to manuscript revision. All authors contributed to the article and approved the submitted version.

## Conflict of Interest

The authors declare that the research was conducted in the absence of any commercial or financial relationships that could be construed as a potential conflict of interest.

## Publisher's Note

All claims expressed in this article are solely those of the authors and do not necessarily represent those of their affiliated organizations, or those of the publisher, the editors and the reviewers. Any product that may be evaluated in this article, or claim that may be made by its manufacturer, is not guaranteed or endorsed by the publisher.
